# Specialist and primary care physicians’ views on barriers to adequate preparation of patients for renal replacement therapy: a qualitative study

**DOI:** 10.1186/s12882-015-0020-x

**Published:** 2015-03-28

**Authors:** Raquel C Greer, Jessica M Ameling, Kerri L Cavanaugh, Bernard G Jaar, Vanessa Grubbs, Carrie E Andrews, Patti Ephraim, Neil R Powe, Julia Lewis, Ebele Umeukeje, Luis Gimenez, Sam James, L Ebony Boulware

**Affiliations:** Johns Hopkins Medical Institutions, Baltimore, MD USA; Vanderbilt University School of Medicine, Nashville, TN USA; Nephrology Center of Maryland, Baltimore, MD USA; University of California, San Francisco School of Medicine, San Francisco, CA USA; Division of General Internal Medicine, Duke University School of Medicine, Durham, NC USA

**Keywords:** Renal replacement therapy, Dialysis, Transplantation, Preparation, Primary care, Collaborative care

## Abstract

**Background:**

Early preparation for renal replacement therapy (RRT) is recommended for patients with advanced chronic kidney disease (CKD), yet many patients initiate RRT urgently and/or are inadequately prepared.

**Methods:**

We conducted audio-recorded, qualitative, directed telephone interviews of nephrology health care providers (n = 10, nephrologists, physician assistants, and nurses) and primary care physicians (PCPs, n = 4) to identify modifiable challenges to optimal RRT preparation to inform future interventions. We recruited providers from public safety-net hospital-based and community-based nephrology and primary care practices. We asked providers open-ended questions to assess their perceived challenges and their views on the role of PCPs and nephrologist-PCP collaboration in patients’ RRT preparation. Two independent and trained abstractors coded transcribed audio-recorded interviews and identified major themes.

**Results:**

Nephrology providers identified several factors contributing to patients’ suboptimal RRT preparation, including health system resources (e.g., limited time for preparation, referral process delays, and poorly integrated nephrology and primary care), provider skills (e.g., their difficulty explaining CKD to patients), and patient attitudes and cultural differences (e.g., their poor understanding and acceptance of their CKD and its treatment options, their low perceived urgency for RRT preparation; their negative perceptions about RRT, lack of trust, or language differences). PCPs desired more involvement in preparation to ensure RRT transitions could be as “smooth as possible”, including providing patients with emotional support, helping patients weigh RRT options, and affirming nephrologist recommendations. Both nephrology providers and PCPs desired improved collaboration, including better information exchange and delineation of roles during the RRT preparation process.

**Conclusions:**

Nephrology and primary care providers identified health system resources, provider skills, and patient attitudes and cultural differences as challenges to patients’ optimal RRT preparation. Interventions to improve these factors may improve patients’ preparation and initiation of optimal RRTs.

## Background

Among the approximately 100,000 patients who developed end-stage renal disease (ESRD) in the United States in 2012, the overwhelming majority (89%) initiated renal replacement therapy (RRT) on hemodialysis [1]. Few patients initiated RRT with self-care dialysis (8.3%) or received a preemptive kidney transplant (2.5%) [1]. Over half (61%) of patients initiating hemodialysis started treatment with a catheter for vascular access [1]. Low rates of self-care dialysis and suboptimal vascular access at initiation may both be related to patients’ lack of timely preparation for renal replacement therapy (RRT).

Despite clinical practice guidelines recommending patients’ timely preparation for RRT, many patients with access to care continue to initiate RRT sub-optimally prepared [1]. Prior to developing ESRD, many patients with advanced chronic kidney disease (CKD) have a poor understanding of their CKD diagnosis and its impact on their health [2,3]. At the time of RRT initiation, many patients are inadequately informed about the various types of available RRT, their potential risks and benefits, or the potential impact these treatments could have on their lives [2,4-8]. Patients’ suboptimal preparation for RRT contributes to their emergent RRT initiation, use of a catheter for vascular access at initiation of hemodialysis, lower utilization of self-care dialysis or kidney transplantation, and increased morbidity (i.e., infections and hospitalization) and mortality [9-14].

Both patient and health care provider factors likely contribute to patients’ suboptimal RRT preparation. Most studies exploring barriers to RRT preparation have focused on patients’ and their families’ experiences with RRT initiation. These studies have identified numerous opportunities to improve patients’ RRT preparation experiences, including improving patient education, providing more time for patients to digest their diagnosis and understanding of their treatment options, and engaging patients’ family members more meaningfully during the RRT preparation process [2,4,6-8]. In contrast, health care providers’ experiences with the RRT preparation process and their perceived barriers to preparation have been less well characterized. Poor preparation is often attributed in part to primary care providers’ late referrals of patients to nephrology care, yet many patients who receive timely referral to nephrologist care are still inadequately prepared [1,15]. Early collaborative and multidisciplinary care models (including partnerships between patients’ primary care and nephrology providers) have been advocated as a health care provider-targeted intervention to improve patients’ CKD care and advanced RRT preparation [16-21], but little is known about factors which may influence providers’ collaborative engagement in RRT preparation.

Investigation of health care providers’ experiences with RRT preparation could provide needed insight into mechanisms through which RRT preparation and patient outcomes can be improved. We performed qualitative directed interviews of nephrology and primary care providers to identify modifiable patient, provider and system-level barriers they face to adequately preparing patients for RRT that could be targeted for future interventions.

## Methods

### Overview

We conducted a qualitative study (directed interviews) of nephrology and primary care providers to inform the development of interventions to address barriers to patients’ optimal RRT preparation. We sought to (1) identify nephrology providers’ perceptions of system, provider, and patient level challenges to RRT preparation; (2) characterize both nephrology and primary care providers’ opinions regarding how the RRT preparation process could be enhanced through engagement of primary care providers; and (3) understand whether perceived barriers to RRT optimal preparation would vary by providers’ practice settings. We recruited the nephrology and primary care providers from two geographically distinct metropolitan areas and in two different types of practice settings.

### Study participants

We recruited a convenience sample of nephrology providers (nephrologists, physician assistants, and a nurse) from two nephrology practices, including a private practice affiliated with a community-based academic hospital in Baltimore, Maryland serving a diverse patient population (including 52% African American) and a public safety-net hospital-based clinic in San Francisco, California serving largely an ethnic and racial minority population (including 25% African American, 25% Hispanic, and 25% Asian). A study investigator at each of the nephrology sites identified the medical director and also provided the names of 2 nephrologists, 2 other nephrology providers (e.g. physician assistant, nurse practitioner, or registered nurse), and 2 primary care physicians who routinely referred to their nephrology clinic site. The recruited primary care physicians also represented a community-based clinic and a public safety-net hospital-based clinic. All of the individuals were invited to participate by phone or via email. We obtained verbal and/or written consent from all study participants. The Johns Hopkins Medicine and University of California, San Francisco Institutional Review Boards approved the study protocol.

### Data collection

The directed interviews were conducted by telephone in July 2012 and August 2012. All interviews were conducted by one investigator (R.G.) with expertise in qualitative research methods [22,23]. Based on findings from prior work [8,24] the study team developed a standard discussion guide to facilitate interviews. Because nephrology and primary care providers have differing roles in the RRT preparation process (with nephrology providers leading the majority of RRT preparation efforts and primary care providers playing a supporting role), we asked them different questions about their experiences with RRT. We asked nephrology providers open-ended questions to assess their perceived challenges to patients’ adequate RRT preparation. We asked both nephrology and primary care providers open-ended questions about their perceived value of nephrologist-primary care provider collaboration in patients’ RRT preparation and challenges they face with collaboration (Table 1). We audio recorded the interviews and transcribed them verbatim.

**Table 1:** **Directed interview questions**

**Nephrology providers**	**Primary care providers**
**Challenges to RRT preparation**	
• What are the biggest challenges you face in preparing patients for renal replacement therapy?	N/A
• What factors make it difficult or easy for you to support your patients’ decision making?	
**Experience with collaborative preparation**	
**•** Please describe your experience with collaborating with patients’ primary care providers?	**•** For your patients who are also cared for by a nephrologist, what has been your experience with that nephrology practice regarding receiving communication about patients’ preparation for renal replacement therapy?
**•** What makes it difficult or easy for you to collaborate with primary care providers?	**•** How involved are you with helping patients prepare for renal replacement therapy?
	**•** How do you see your role?
	**•** Would you like to be more involved? If yes, how would you like to be involved?

### Analysis

We used the grounded theory approach for content analysis [25,26]. Using the constant comparative method, two abstractors (JA, CA) independently reviewed the interview transcripts to iteratively develop a coding scheme representing the relevant concepts voiced in the interview discussions. The investigators then categorized codes to create a list of key themes and arrived at a consensus on a final list of themes that emerged during the interviews pertaining to providers’ perceptions of system, provider and patient challenges associated with the RRT preparation process. We subsequently also reviewed themes to determine if differences emerged based on providers’ practice types.

## Results

A total of 14 providers were invited and agreed to participate in the directed interviews, including 10 nephrology providers [nephrologists (n = 6), physician assistants (n = 3), and a registered nurse (n = 1)] and four primary care physicians. The interview duration for nephrology providers ranged from 40-67 minutes in length (average length: 51 minutes) and for primary care providers ranged from 14-42 minutes in length (average length: 24 minutes).

### Nephrology providers’ perceived challenges to patients’ adequate RRT preparation

Nephrology providers identified several system, provider, and patient-level challenges to preparing patients for RRT that encompassed 9 themes (Table 2).

**Table 2:** **Providers’ perceived challenges to patients’ preparation for renal replacement therapy**

**Themes**	**Subthemes**	**Type of providers identifying**
**System**-**level challenges**		
Limited time for optimal patient preparation	**•** Limited time to prepare	Nephrology
	**•** Limited time to build trusting relationships	Nephrology
Referral process delays	**•** Delays in referral to vascular surgery	Nephrology
Poor primary care/nephrology co-management	**•** Poor information exchange	Nephrology and Primary care
	**•** Lack of patient CKD education prior to nephrology referral	Nephrology
	**•** Poor delineation of roles	Primary care
**Provider**-**level challenges**		
Provider difficulty with explaining CKD and confirming patient understanding	**•** Conveying CKD in lay terms	Nephrology
	**•** Uncertainty about patient understanding of RRT options	Nephrology
**Patient**-**level challenges**		
Patients’ poor acceptance and understanding of CKD	**•** Denial of CKD diagnosis and/or severity	Nephrology
	• Poor understanding of CKD	Nephrology
	• Low awareness/understanding of treatment options	Nephrology
Patients’ low perceived urgency for RRT preparation	**•** Delayed preparation because asymptomatic	Nephrology
	**•** Poor compliance with RRT preparation appointments	Nephrology
Patients’ negative perceptions about RRT	**•** Fear/anxiety about dialysis	Nephrology
Patient cultural or language differences	**•** Patient preference for alternative treatments	Nephrology
	**•** Poor patient understanding due to cultural and/or language differences	Nephrology
Lack of patient trust	**•** Low patient trust in health providers	Nephrology

## System level challenges

### Nephrology providers’ limited time

Most nephrology providers felt that they did not have adequate time to optimally prepare patients for RRT. When confronted with challenging patients, such as those who are in denial of the severity of their CKD or who refuse treatment due to negative perceptions of RRT, nephrology providers often felt that there wasn’t sufficient time to build a trusting relationship with the patient. They reported that often patients were referred so late that some patients did not have an opportunity to comprehend their new CKD diagnosis prior to being asked to prepare for RRT. The limited time also restricted the treatment options that were available to the patient. A nephrology provider commented:

*“…there’s not enough time to build a solid relationship …with the patient, before the idea of renal replacement even comes up and so partly that results in many catheter starts…instead of fistula graft starts and really an inability to refer for preemptive transplant often.”(Physician)*

Many nephrology providers attributed this limited time to late referrals from primary care providers or limited care and/or access to care prior to ESRD.

### Nephrology providers’ difficulty obtaining vascular access referrals

Nephrology providers reported that, even when they felt that they had reached a decision with the patient on a RRT option, the preparation process was often delayed due to difficulty with obtaining a timely specialty appointment for vascular access placement. Nephrology providers reported that, in some cases, delays for vascular access were related to health insurance administrative issues, such as the need to get prior authorization for the visit. A nephrology provider commented:

*“…it’s becoming increasingly difficult to refer them out to get their vascular accesses placed… [It’s] becoming a little more complicated with trying to get prior authorizations and so on.”(Physician)*

### Poorly integrated primary and nephrology care

Nephrology providers identified several challenges to co-management of patients with advanced CKD, including poor information exchange, late timing of nephrology referral by primary care providers and lack of patient education about CKD prior to the nephrology referral. Some nephrologists felt that collaboration with primary care providers was minimal and found it challenging that they didn’t regularly receive the primary care providers notes to update them on interval medical issues and adjustments in patients’ chronic disease management. A nephrologist reported, “…it’s a very cordial working relationship, but there’s actually usually not a lot of communication.…” Another nephrologist commented,

*“One disappointing thing is that the average rate of communication the other way around for me to them is 100*% *in my patients. Every single note that I generate goes to the primary care whether I like it or not, getting notes from primary care doctors is less than 10*%*, maybe less than 5*%*, and that’s bad because that is a big source of miscommunication, things not done properly or whatever so that’s my concern.”(Physician)*

Many nephrologists were frustrated that often patients were unaware of their kidney disease and its health implications prior to the initial nephrology visit.

A nephrologist stated:

*“I would just want to have primary care providers understand the dilemma that we get into when referrals come late….it’s just so much more distressing for the patient because a lot of the late referrals they’ve either never been told anything about the fact they have kidney trouble or they have but it’s been so downplayed and pushed aside that the actual severity of it is completely shocking to the patient once we talk to them about it.”(Physician)*

Primary care providers also identified several challenges to successful co-management of patients with advanced CKD. While the primary care providers in our study generally felt that they were informed of the nephrologists’ evaluation and management plan via receipt of the consultative note, one primary care provider reported receiving little communication from nephrology, especially during the time period when patients are approaching the need for renal replacement therapy: “There is very little communication. Usually once we send patients who are going to need dialysis…it’s a struggle to get communication back and forth”. A number of primary care providers also felt that once they referred patients to nephrology, the nephrologists “generally [take] over the management of the patient,” with poor delineation of roles or even lack of a role for the primary care provider in the care of the patient. One primary care provider noted: “…their notes are comprehensive, but they don’t have a role for me.” Another primary care provider commented,

*“I would like [to get] the sense more of teamwork….I would like to be thought of and operate as a respected colleague who actually probably knows the patient much, much better and will see the patient quite frequently. That one doesn’t always have the sense that is the attitude of many such specialists, including many of the nephrologists” (Physician)*

Some primary care providers desired more involvement in preparing patients for RRT and ensuring smooth RRT transitions. Due to their long-standing relationship with the patient, primary care providers felt that they often play a key role in helping patients make treatment choices. A primary care provider commented:

*“Well, I think we’re there as a helpful role to the patients because you know often we’ve had a long relationship with the patient and they trust our opinions. So I think, you know, we do play a big role in ultimately helping them make their decision…” (Physician)*

Most primary care providers saw their role during the RRT preparation process as providing patients with emotional support, helping patients understand and weigh RRT options, affirming the nephrologists’ RRT recommendations, and confirming patients’ certainty about their modality choice. In addition, they felt they could help facilitate patients’ smooth transition to RRT, including patients’ completion of necessary consultations, tests and procedures. A primary care provider stated:

*“Well I think my role obviously is first of all to try to prevent [ESRD] from happening in the first place. But my role [related to RRT preparation] is to try to help the transition be as smooth a one as possible to help the patients have as few complications as possible and to help support the relationship that they have with the renal service and then eventually with dialysis.”(Physician)*

## Provider level challenges

### Nephrology providers’ difficulties explaining CKD and confirming patients’ understanding of CKD

Some nephrology providers reported they experienced difficulty getting patients to understand what the kidneys do and to “fully appreciate” the severity of their CKD and the importance of preparation, particularly when educating patients with low education levels. In addition, several nephrology providers were not confident in their ability to elicit patients’ concerns about their CKD and the treatment options. A nephrology provider stated:

*“…there might be something that you’re not asking that….they’re not telling you, so you can’t answer the question in order to help them make a good decision.”(Physician Assistant)*

## Patient-level challenges

### Patients’ poor understanding and acceptance of CKD

Nephrology providers frequently identified patients’ difficulty accepting and understanding their CKD diagnosis and recognizing the impact of CKD on their future health as a challenge. Providers reported they believed CKD was hard for patients to understand and that patients had difficulty fully comprehending their treatment options and the different treatment experiences. One nephrology provider commented, “Patients don’t have a good handle on what dialysis is until they are on it…” Another stated “…you know the kidneys are not the easiest thing for a lay person to understand. It’s a little hard to sort of get the message to them that this actually is important”. Providers also noted they often spend time addressing patients’ misconceptions and that there is a lack of “any common understanding” of chronic kidney disease and its treatment. Many nephrology providers also noted patients’ denial of the severity of their CKD as a significant barrier to initiating RRT preparation. One nephrology provider stated,

*“They do see so many of their family members going through the same sorts of things that sometimes they just kind of want to stick their heads in the sand and ignore that maybe that’s going to also happen to them.”(Physician Assistant)*

Another nephrology provider commented,

*“… [Some patients] will not allow me to initiate dialysis because they feel uncomfortable. They don’t think it’s necessary even though I try to explain why to them, and it’s hard for me sometimes to break the barrier. I don’t know how to address that…” (Physician)*

### Patients’ low perceived urgency for RRT preparation

Many nephrology providers believed that because patients are typically asymptomatic prior to the need for RRT initiation, patients feel that preparation is “really not that imperative”. Some nephrology providers reflected that, due to this perceived lack of urgency, many patients don’t complete the steps to prepare for RRT in a timely fashion, including non-compliance with surgical visits for vascular access and/or educational classes. One nephrology provider stated:

*“The other thing that’s difficult is people that say I’m just going to wait until I’m sick; I feel fine now. Trying to get them to agree…to choose a modality and then make the preparations in advance is very difficult.”(Physician Assistant)*

Additionally, some nephrology providers described that patients were concerned that early preparation may lead them to initiate renal replacement therapy earlier than necessary. A provider commented:

*“…people think if they prepare and get a fistula placed, they’re going to need dialysis sooner, like they’re on the track versus if they just avoid it maybe they won’t need it as soon.”(Physician Assistant)*

### Patients’ fear of dialysis

Many nephrology providers reported patients’ fear of dialysis as a challenge to optimal patient preparation. Nephrology providers related that many patients know someone (e.g., family, friends, or neighbors) who has been treated with dialysis and experienced a poor outcome. They relate that they feel patients are very concerned about experiencing a similar fate. A nephrology provider commented:

*“…there seems to be this stigma attached to it that is once you start bringing up dialysis, it’s over. They may as well start getting their will together, and it’s just sort of a terminal pathway, and one thing I hear a lot is oh no, no, no because you know I knew this guy and I knew this woman who they went to dialysis, and a month later they were dead.”(Physician)*

### Patients’ cultural beliefs or language differences

A number of nephrology providers experienced challenges to RRT preparation when patients’ cultural and spiritual beliefs conflicted with their clinical recommendations. Nephrology providers commonly cited patients’ preferences for alternative treatments (e.g., herbal remedies) to treat kidney disease as a barrier to their care as well as patients’ beliefs that “medications are bad in general and anything to do with…that sort of care [western medicine] is bad”. One nephrology provider commented:

*“We do have some patients taking herbal preparations that have been found to be damaging to their kidneys and it’s really difficult to convey respect for their culture and that approach, at the same time saying it’s damaging and it’s not helping you.”(Physician Assistant)*

Language differences were also identified as another barrier to patients’ RRT preparation.

### Patients’ lack of trust

Some nephrology providers also cited patients’ low trust in health care providers and the health system as a challenge to RRT preparation, particularly among their African American patients. Nephrology providers relayed they felt patients were concerned that they were being experimented on and were not being offered the standard of care. One nephrology provider commented,

*“I’m certain many of them don’t trust us, what we are saying to them. They don’t feel as sick as we say they are. So they just don’t feel inclined to do these things ahead of time.”(Physician)*

### Variation in provider insight based on clinical practice type

Similar challenges to optimally preparing patients for RRT were identified among nephrology providers at both practice settings (i.e., private practice affiliated with community-based academic hospital and at the patient safety-net hospital-based clinic). However, language barriers and preference for alternative therapies were uniquely identified themes reported by providers at the patient safety-net hospital-based clinic serving a large immigrant population. Poor information exchange was mostly reported by nephrologists who did not share an electronic medical record with their referring primary care provider.

## Discussion

Nephrology and primary care providers identified several system (including limited time for patient preparation, referral process delays, and poorly integrated nephrology and primary care), provider (including providers’ difficulty with explaining CKD and confirming patient understanding), and patient (including patients’ poor acceptance and understanding of their CKD and its treatment options, low perceived urgency for RRT preparation, negative perceptions about RRT, cultural or language differences, and lack of trust) challenges contributing to patients’ suboptimal RRT preparation. Insights were largely similar in two different practice settings, although providers noted cultural and language differences as distinct challenges in the public safety net setting. Findings reinforce ongoing efforts to improve RRT education, and they lend new insight to numerous additional targets for future interventions to improve RRT preparation.

Prior studies describing challenges with RRT preparation have focused mostly on patients’ and their families’ perceptions of RRT preparation but they have not centered on providers’ views. Patients and their families have previously described suboptimal experiences with RRT preparation, stemming from their poor understanding of kidney disease prior to developing ESRD, their urgent initiation of dialysis without adequate preparation or education, and poor support for their decision-making after dialysis initiation [4,6,8,27]. Suboptimal discussion of treatment alternatives and outcomes, as well as suboptimal encouragement of transplantation particularly among patients with lower socioeconomic status have also been identified by patients and their nephrology providers as barriers to patients’ access to various RRTs [5,7,28]. Although, some of the issues raised by providers are similar to those raised by patients, our findings on providers’ views extend this prior work by identifying potential resources and skills providers may need to improve preparation for RRT in their practices, by providing insight into ways collaboration could help improve RRT preparation, and by exploring how providers’ needs for interventions to improve RRT preparation might vary in different practice settings.

Providers’ views not only reinforced the widely recognized need for enhanced resources to adequately educate patients about kidney disease [17,29], but they also highlighted the need for additional types of resources that could help them overcome patient challenges to RRT preparation. For instance, providers identified a need to help patients combat their denial of kidney disease progression, as well as to help patients confront negative perceptions of RRT and to overcome their psychological avoidance of RRT. These findings are supported by those from a single Canadian study which identified patient-level delays, such as patients’ hesitation to receive education or to consider vascular access, and their lack of adherence to nephrologist recommendations to pre-dialysis care, as barriers to suboptimal RRT preparation [30]. Interventions that more readily expose patients to typical experiences of other patients with kidney disease and resources to help patients navigate the RRT preparation process could help patients overcome these challenges to RRT. Peer-led support (such as patient navigators) for adapting to major life events such as cancer diagnoses and treatment have been shown to help patients adjust to their diagnoses and subsequent cancer care [31,32]. Similar efforts to better expose patients to others’ experiences with advanced kidney disease and on various RRTs could help clarify for patients the importance of early engagement in RRT preparation as well as to demystify their concerns about the treatment options. Patient navigators have also been employed to help patients make complex medical decisions including pursuit of kidney transplantation [33], and have been shown to be beneficial in improving patients’ self-management of chronic conditions, including diabetes [34]. Efforts to engage peers or lay health educators of similar background or cultural experiences to help patients navigate the RRT preparation process (e.g., decision-making and completion of RRT preparation steps) as well as the provision of language and culturally appropriate materials could help patients establish greater trust and confidence in the RRT preparation process.

Providers also identified several of their own challenges to RRT preparation, some of which have been previously identified [2,6,15]. These include their difficulties with establishing working partnerships with patients as well as difficulties eliciting patient concerns and confirming patient understanding about treatment options. Nephrology providers may need advanced communication skills and cultural competency training to help them establish better partnerships during RRT preparation and increase patients’ autonomous motivation to prepare for RRT. Specifically, they may need skills related to shared decision making or motivational interviewing to (1) empathically but effectively break the news to patients that they will likely need RRT, (2) ensure patients understand the risks and benefits of all their treatment options, (3) support patients’ choices of RRT that align well with patients’ personal values, and 4) resolve patient barriers such as denial or ambivalence that contribute to delays in RRT preparation. Given the often protracted nature of kidney disease progression, nephrology providers may need to employ skills in these areas repeatedly over time. Early interventions to improve patient-provider communication to enhance shared and informed decision-making on RRT are being developed [35], but they have not yet been integrated into routine clinical practice. In other chronic conditions, efforts to increase the implementation of shared decision making and/or motivational interviewing in clinical practice have been shown to improve patients’ self-confidence in approaching changes in treatment management, and to improve patients’ engagement in care, risk factor management, and achievement of informed, values-based treatment choices [36-38]. Programs incorporating shared decision making and motivational interviewing principles to help nephrology providers gain these skills (e.g., during nephrology fellowship training) could substantially improve the degree to which providers feel enabled to help patients better prepare for RRT.

Nephrology providers noted limited advance time to prepare patients for RRT due to late referrals from primary care providers as a major system level challenge to RRT preparation. Clinical practice guidelines recommend referral to subspecialty care among patients with advanced (estimated glomerular filtration rate <30 ml/min/1.73 m^2^) and/or progressive CKD to afford sufficient time to plan and prepare for RRT [39-41]. Primary care providers late referrals, as well as limited patient-physician discussions about CKD prior to the nephrology visit likely stem from numerous factors including their uncertainties about when to refer patients [42,43], visit time constraints, fears of overwhelming patients with news of kidney disease, and their lack of self-efficacy with educating patients about CKD [23]. Efforts to better educate primary care providers on kidney disease treatment and referral guidelines, to enhance patient education about CKD in primary care, and to enhance collaborative and coordinated care between nephrology and primary care providers could enhance primary care providers capacity to support patients [44]. Consistent with published guidelines on care coordination [45], nephrology-primary care partnerships that 1) establish accountability or negotiate responsibility for RRT preparation, 2) foster frequent communication about RRT preparation and facilitate transitions between primary care and nephrology care, and 3) clearly establish the goals of RRT preparation efforts may be most effective. Given the significant overlap in care responsibilities between primary care and nephrology providers, these care coordination activities become especially important to facilitating patients’ smooth transition to RRT. Early strategies to improve primary care provider/nephrologist collaboration (i.e., in the form of more comprehensive consultation letters from nephrology to primary care providers) have been developed, but evidence regarding their effectiveness when implemented in practice is limited [46,47]. Likewise, primary care provider letters to nephrologists have also not been studied.

Our identification of similar as well as distinct challenges in different practices highlights the importance of accounting for differences in patient populations that could affect barriers faced to a coordinated and timely transition to RRT. For example, access to interpreters as well as linguistically and culturally appropriate educational materials that are clear, easy to use, and written in plain language is essential for practices serving diverse patient populations. Additionally, enhanced use of clinical information systems that can be shared across health systems is needed to facilitate information exchange between patients’ multiple health care providers to improve care coordination during the often multifaceted RRT preparation process.

Our study has limitations. First, while we believe our findings are reflective of providers’ perspectives of common challenges to RRT preparation across the United States, we interviewed only a few providers at each site in this small qualitative study. It is possible that providers in other geographic or practice settings might identify additional or different challenges to RRT preparation. Second, while our providers identified numerous types of challenges to RRT preparation, we did not ascertain which challenges they more frequently confront or those challenges most likely to hinder optimal RRT preparation.

## Conclusions

In conclusion, nephrology and primary care providers identified system, provider and patient challenges to RRT preparation. Interventions designed to help patients better understand and adjust their expectations regarding RRT preparation, to help nephrology and primary care providers gain skills to support preparation, and to enhance collaboration between nephrology and primary care providers could help patients establish more successful transitions to RRT. Practices with different patient populations and structures may need additional resources to address patients’ culture or language needs and to facilitate better information transfer between providers during RRT preparation.
